# Prevalence and Genotyping of* Echinococcus* Species from Livestock in Kajiado County, Kenya

**DOI:** 10.1155/2019/4798906

**Published:** 2019-07-07

**Authors:** Lucy Nungari, Cecilia Mbae, Joseph Gikunju, Erastus Mulinge, Timothy Kaburu, Eberhard Zeyhle, Japhet Magambo

**Affiliations:** ^1^Kenya Medical Research Institute, Nairobi, Kenya; ^2^College of Health Sciences (COHES), Jomo Kenyatta University of Agriculture and Technology, Nairobi, Kenya; ^3^Meru University of Science and Technology, Meru, Kenya

## Abstract

Cystic Echinococcosis (CE) is a widespread neglected zoonotic disease and is caused by the larval stage of the dog tapeworm* Echinococcus granulosus *sensu lato. CE is more frequent in livestock-rearing areas and where people live a nomadic or seminomadic lifestyle such as in Kajiado County, Kenya. There is limited data on CE disease situation in the county of Maasailand; the present study, therefore, reports on the prevalence of CE in cattle, sheep, and goats and their relative importance in CE transmission in Kajiado County. In total, 1,486 livestock (388 cattle, 625 sheep, and 473 goats) slaughtered in two abattoirs were examined for the presence of hydatid cysts in various organs. Cyst isolates were genotyped by polymerase chain reaction-restriction fragment length polymorphism (PCR-RFLP) of the NADH dehydrogenase subunit 1 gene (*nad1*). The overall prevalence of CE was 14.8% (220/1486), while prevalence per livestock species was 15.2% (72/473) in goats, 14.9% (93/625) in sheep, and 14.2% (55/388) in cattle. Out of the 421 cysts isolated, 389 cysts were successfully characterized to be either* E. granulosus *sensu stricto (s. s.), 356/389 (91.5%),* E. canadensis* (G6/7), 26/389 (6.7%), or* E. ortleppi*, 7/389 (1.8%). This record confirms predominance of* E. granulosus* s. s. in Maasailand and other parts of Kenya, while the importance of* E. ortleppi* and* E. canadensis* (G6/7) to the general CE burden in Maasailand might be higher than previously thought. More so, a higher infection pressure for humans by* E. granulosus* s. s. based on its abundance could be speculated. The study sheds significant light on CE situation in livestock in the nomadic/seminomadic society of the Maasai in Kajiado County and provides good bases to investigate human CE in the area.

## 1. Introduction

Cystic Echinococcosis is caused by the larval stage of the dog tapeworm* Echinococcus granulosus *sensu lato (s. l.) and is currently recognized by World Health Organization (WHO) as a neglected tropical disease [1]. CE is a common zoonotic disease of great public health significance globally due to its associated economic losses [2]. Approximately US$ 3 billion are lost annually on treatment of CE in humans and losses incurred due to the condemnation of infected organs in livestock [3]. Dogs and to a lesser extent other canids and felids are the primary definitive hosts of* Echinococcus* species, with herbivores acting as the intermediate hosts and the humans as aberrant intermediate hosts. The outcome of the infection in livestock and human is cyst development in the liver, lungs, or other organs [4].* E. granulosus* s. l. consists of at least five species, namely,* E. granulosus *sensu stricto (s. s.),* E. equinus, E. ortleppi, E. canadensis* (G6–G10), and* E. felidis *[5, 6].

East Africa and Kenya, in particular, have long been known to be of the world's largest foci of CE in humans [7–9]. Previous data from Kenya have centered their focus on CE situations in Turkana and Maasailand [8]. Data from other endemic areas are available but only sparingly. Previous CE studies in Kenya, thus far, indicate the presence of all five* E. granulosus *s. l. and the recently discovered Gomo genotype [10–15]. To appreciate the CE situation in the whole of Kenya, epidemiological data from all endemic localities including Kajiado County is required. The only available data from this area is nearly three decades old and did not report* Echinococcus* spp. in livestock [16]. Furthermore, the recent study examined livestock originating mainly from Bissil area (Kajiado South) [10]. Therefore, this study focused on the two main slaughterhouses receiving livestock from the wider scope of the Kajiado County. We report here the prevalence of CE in cattle, sheep, and goats and the* Echinococcus* spp. causing CE in Kajiado County. Findings from the study will improve our knowledge of CE in this county and establish the relative contribution of each livestock species in the distribution and transmission of CE.

## 2. Materials and Methods

### 2.1. Hydatid Cysts Collection from Cattle, Sheep, and Goats

**Figure 1:**
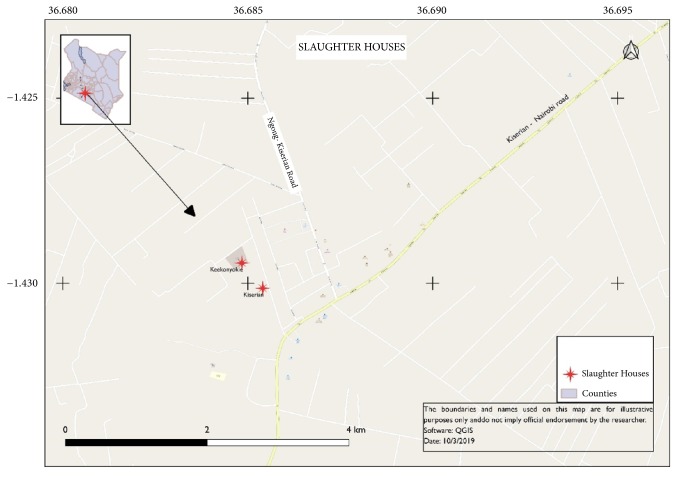
A map showing the location of the two abattoirs located in Kiserian and Keekonyokie.

Majority of the livestock examined for CE came from Kajiado County. The county is divided into five subcounties: Kajiado Central, Kajiado North, Kajiado East, Kajiado West, and Kajiado South. Sampling was done in two major abattoirs in Kajiado West Subcounty, namely, Kiserian and Keekonyokie, during slaughter days from December 2016 to February 2017 (Figure 1). A total of 1486 carcasses of livestock were inspected for the presence of hydatid cysts in all organs (lungs, liver, heart, spleen, as well as the kidneys) of the pleural and abdominal cavities. Visual inspection, palpation, and incision were done for all of the organs for the presence and cyst distribution. The lesions were carefully excised from all infected organs. Individual cysts were identified as those that had a continuous cyst wall while multiple cysts had a visibly separate cyst wall even for the calcified cysts. The isolated cysts were packed in clean polythene bags placed in cooler boxes and transported to the parasitology laboratory of the Kenya Medical Research Institute, for examination and further analysis. Cysts were dissected using a sterile scalpel blade and each cyst material was fixed and preserved in 70% Ethanol in individual tubes. The contents of the cysts were examined microscopically for the presence of protoscoleces (PS). Cysts were classified as fertile (with protoscoleces) sterile (fluid-filled without protoscoleces), degenerated (collapsed cyst walls with caseated protoscoleces and soft cheesy debris without calcification), and calcified (hard solid appearance of the ectocyst). All the cysts from the same organ were examined individually to confirm mixed infections.

### 2.2. DNA Extraction

DNA was obtained from cyst material and protoscoleces by lysing in 0.02 M NaOH at 99°C for 10 minutes. In a few instances where the above process failed to yield adequate DNA, genomic DNA was extracted using DNeasy Blood & Tissue Kit® (Qiagen, Hilden, Germany). The germinal layers or cyst walls were cut into small pieces and lysed in ATL lysis buffer (180 *μ*l) and proteinase K (20 *μ*l), and DNA was subsequently extracted using the manufacturer's protocol. Extracted DNA was eluted in 50 *μ*L of elution buffer.

### 2.3. Polymerase Chain Reaction and Restriction Fragment Length Polymorphism (PCR-RFLP)

Two nested PCR assays targeting part or the entire NADH dehydrogenase subunit 1 gene (*nad1*) were used for genotyping of cyst materials. The first nested PCR (entire* nad1 *gene) was performed as described by Hüttner and Nakao [17]. The cyst materials negative using the first PCR assay were genotyped using a second nested PCR as described by Mulinge and Magambo [18], which amplifies part of the* nad1* gene (545-552 bp). In both PCR assays, the reaction mixture contained 2 *μ*l of the DNA, 1 × DreamTaq Green Buffer (20 mM Tris-HCl (pH 8.0), 1 mM DTT, 0.1 mM EDTA, 100 mM KCl, 0.5 % (v/v) Nonidet P40, 0.5 % (v/v) Tween 20) (Thermo Scientific), 0.2 mM dNTPs, 0.25 *μ*M of forward and reverse primers, 2 mM MgCl_2_, and 0.625 units of DreamTaq Green DNA Polymerase (Thermo Scientific) in 25 *μ*l final volume. The PCR cycling conditions were 5 min. for initial denaturation at 94°C, 40 cycles of 94°C for 30 s, 55°C for 30 s and 72°C for 60 s, and a final extension at 72°C for 5 min. Positive PCR products were genotyped by RFLP to the specific* Echinococcus *species. To this end, 10 *μ*l of the nested PCR products was digested using 0.5 *μ*l (5 U) of* Hph*I restriction enzyme, 1 × Buffer, and 7.5 *μ*l of nuclease-free water and incubated at 37°C overnight [18, 19]. Positive controls for* E. granulosus *s. s.,* E. ortleppi, E. canadensis *(G6/7), and* E. felidis were* resolved alongside the test samples for both methods.

### 2.4. Ethical Approval

Institutional approval was granted by the Institute of Tropical Medicine and Infectious Diseases (ITROMID) at the Jomo Kenyatta University of Agriculture and Technology (JKUAT). The study protocol received ethical clearance from the Department of Veterinary Services, Kajiado County, and by KEMRI's Scientific Ethics Review Unit (SERU) (P00048/3395) and Animal Care and Use Committee.

## 3. Results

### 3.1. Prevalence, Cysts Location, Load, and Conditions

A total of 1486 livestock at slaughter were screened during the survey. The general prevalence of CE in the current study area was 14.80% (220/1486): more common in goats 15.22% (72/473) than in sheep 14.88% (93/625) and in cattle 14.18% (55/388) (Table 1). In all the infected livestock, liver and lungs were the only organs harbouring cysts, and the liver was the most infected organ (*p = 0.013)*. Across infected livestock, 421 cysts were isolated: 260 (61.76%) were from the liver and 161 (38.24%) from the lungs (Table 1). Though CE infection in goat was the highest, infections with more than one cyst were higher in cattle and sheep than in goat (Table 2). Majority of cysts of cattle origin were sterile (41.9%), while most of the calcified cysts were found in sheep (48.5%) and goats (52.2%) (Tables 3 and 4). On average, sheep had the greatest number of fertile cysts at 40.5%, 25.9% in cattle and 18.3% in goats (Tables 3 and 4).

### 3.2. Genotyping

From a total of 421 cysts that were subjected to nested PCR-RFLP, 389 cysts were successfully genotyped and included 137/143 (95.8%) from cattle, 103/115 (89.57%) from goats, and 149/163 (91.4%) from sheep. A total of 32/421 cysts failed to amplify, thus, 6, 12, and 14 from cattle, goats, and sheep, respectively, and therefore were not characterized (Table 4). Three species of* Echinococcus* were identified and included* E. granulosus* s. s. 356 (91.5%),* E. canadensis* (G6/7) 26 (6.7%), and* E. ortleppi *7 (1.8%).* E. canadensis* (G6/7) infection was higher in goats 17 (16.5%) than in cattle 2 (1.5%) and sheep 7 (4.7%). All the fertile cysts in cattle (25.9%), 18.3% in goats, and 40.5% in sheep except one belonged to* E. granulosus* s. s taxon. The other fertile cyst from sheep was* E. canadensis* (G6/7). All the remaining cysts identified as* E. canadensis* (G6/7) (25) and* E. ortleppi* (7) were not fertile (Table 4).

In addition to single infections, this study reports several cases of mixed infections. There were three cases of mixed infections in cattle and there was one case of all three* Echinococcus *spp. (*E. granulosus *s. s.,* E. ortleppi*, and* E. canadensis *(G6/7) as well as two instances of* E. granulosus* s. s. and* E. ortleppi* and* E. ortleppi *and* E. canadensis* (G6/7). In goats, there were three cases of mixed infections, all with* E. granulosus *s. s. and* E. canadensis* (G6/7), and, in sheep, only one case of* E. granulosus *s. s. and* E. canadensis * (G6/7) mixed infection was observed (Table 5).

## 4. Discussion

This study reports the prevalence of cystic echinococcosis (CE) and* Echinococcus* spp. in cattle, goats, and sheep in Kajiado County, Kenya. The prevalence reported is within the range known of Maasailand from older accounts, such as in the works of Macpherson [16] (8.9%, 8.1%, and 7.1% in cattle, sheep, and goat, respectively) and Addy et al. [19] (25.8 % in cattle, 16.5 % in sheep, and 10.8% in goats). It confirms the persistence of CE in nomadic society. High livestock stocking intensity, conducive environmental conditions, and movement of livestock [20, 21] may have influenced the infection pressure and the persistence of the cestode in livestock intermediate hosts in Maasailand.

The liver was the most affected organ just as was known before from Maasailand [19]. The predilection site of* E. granulosus *s. l. is not fully understood and some studies [16, 20, 22] indicated the lungs to be the most affected. Cysts in the liver or the lungs could be either fertile containing protoscoleces/daughter cysts or nonfertile. The nonfertile cysts can further be divided into calcified, degenerated, or sterile. These nonfertile cysts are noninfectious and, therefore, have no epidemiological significance in CE transmission to definitive hosts. In this study majority of the cysts from livestock were nonfertile, and a recent survey reported 80% of cysts from sheep in Turkana being calcified (Zeyhle unpublished data). This observation is not clearly understood, because regular deworming of ruminants is less likely to have a significant effect on the calcification of cysts. Previous studies have shown that long-term treatment with high doses of anthelmintic drugs is required to arrest cyst development [23, 24]. Sheep in which most fertile cysts were isolated in the present study would be more important in the transmission and maintenance of CE in Maasailand. The cysts fertility rates reported indicate the need for control measures such as health education, regular deworming of dogs, dog population control, good slaughter hygiene, and proper disposal of slaughter offal to avert transmission.

Majority of the cysts in this study were* E. granulosus *s. s. which confirms its predominance observed almost a decade ago in Maasailand [10] and in Kenya at large [11, 13, 15, 25, 26]. The high fertility rates of* E. granulosus* s. s. cysts in sheep indicate that they are important intermediate host of this taxon in this area. Sheep are also the most common home-slaughtered livestock species in Maasailand and that may enhance transmission of* E. granulosus* s. s. However, both cattle and goats may also play a role in transmission of* E. granulosus* s. s. based on the fertility rate in this study. Although goats are considered important intermediate host of* E. canadensis* (G6/7) in absence of camels, none of the cysts belonging to this taxon were fertile in this study [10, 27]. Isolation of* E. ortleppi* from all three livestock species reveals a wider host range of the parasite, aspect that is less understood. Generally,* E. ortleppi *is a rare species even in cattle who are the principal intermediate hosts, possibly due to the fact that cattle are rarely slaughtered at home, and therefore dogs have less access to slaughter offal from cattle [10]. However, in a recent development, due to poor disposal of condemned viscera in poorly managed slaughter facilities in urban centres, dogs have readily access to slaughter offal and this might be a reason for the increased cases of* E. ortleppi *in our study [18]. Elsewhere in Brazil home slaughter of cattle is believed to be a factor that facilitates the recent rise of* E. ortleppi *prevalence in Brazil [28].

## 5. Conclusion

Cystic echinococcosis continues to persist in Maasailand with* E. granulosus *s. s. being the dominant species. The high fertility rate of cysts in sheep and its regular home-slaughter make it the most important intermediate host in the transmission of CE in Kajiado County of Kenya.* Echinococcus ortleppi (G5)* and* E. canadensis* (G6/7) may be important CE agents in Maasailand more than previously thought.

## Figures and Tables

**Table 1:** Prevalence of cystic echinococcosis and cyst location in cattle, goats, and sheep in Kajiado County.

Livestock	Prevalence (%)	Liver	Lungs	Both organs
Cattle (n=388)	55 (14.18)	25	16	14
Goats (n=473)	72 (15.22)	45	21	6
Sheep (n=625)	93 (14.88)	67	18	8

**Table 2:** Cyst load in infected cattle, goats, and sheep in Kajiado County.

	Cysts n (%)				
Livestock	1	2	3	4	5 or More
Cattle (55)	26 (47.3)	14 (25.5)	2 (3.6)	8 (14.5)	5 (9.1)
Goat (72)	51 (70.8)	10 (13.9)	6 (8.3)	3 (4.2)	2 (2.8)
Sheep (93)	64 (68.8)	17 (18.3)	5 (5.4)	1 (1.1)	6 (6.5)

n = number of cysts.

**Table 3:** Condition of isolated cysts from cattle, goats, and sheep in Kajiado County.

Condition of cysts							
Livestock		Fertile	Sterile	Degenerated	Calcified	Total	Fertility rate
Cattle	Liver	7	34	14	22	77	
	Lungs	30	26	6	4	66	
	*Total*	*37*	*60*	*20*	*26*	*143*	*25.9*%
Goat	Liver	14	0	10	51	75	
	Lungs	7	3	21	9	40	
	*Total*	*21*	*3*	*31*	*60*	*115*	*18.3*%
Sheep	Liver	25	4	8	71	108	
	Lungs	41	1	5	8	55	
	*Total*	*66*	*5*	*13*	*79*	*163*	*40.5*%

**Table 4:** Condition and frequencies of *Echinococcus* spp. isolated cysts from cattle, goats, and sheep in Kajiado County.

	Condition of cysts (n (%) and *Echinococcus* spp.			
Livestock	Fertile	Sterile	Degenerated	Calcified
Cattle	37 (25.9%) (37* E. granulosus *s. s.)	60 (41.9%) (52* E. granulosus *s. s*., *3* E. ortleppi, *5 NC)	20 (14%) (19* E. granulosus s. s*., 1* E. canadensis* (G6/7))	26 (18.2%) (23* E. granulosus *s. s*., *1* E. ortleppi, *1* E. canadensis* (G6/7),1 NC)
Goat	21 (18.3%) (21 *E. granulosus *s. s.)	3 (2.6%) (3 *E. granulosus *s. s.)	31 (26.9%) (27 *E. granulosus *s. s*., *4* E. canadensis *(G6/7))	60 (52.2%) (33 *E. granulosus *s. s*., *2* E. ortleppi, *13* E. canadensis *(G6/7), 12 NC)
Sheep	66 (40.5%) (65 *E. granulosus *s. s*.,*1* E. canadensis* (G6/7))	5 (3.1%) (5 *E. granulosus *s. s.)	13 (7.9%) (11 *E. granulosus *s. s*.,*1* E. canadensis* (G6/7), 1 NC)	79 (48.5%) (60 *E. granulosus *s. s*., *1* E. ortleppi, *5* E. canadensis* (G6/7), 13 NC)
*Total*	*124*	*68*	*64*	*165*

n = number of cysts

NC = Not characterised

**Table 5:** Frequency of single or mixed infections in cattle, goats, and sheep in Kajiado County.

	Livestock n (%)		
*Echinococcus* spp.	Cattle	Goat	Sheep
*E. granulosus *s. s.	51 (92.7)	50 (73.5)	79 (90.8)
*E. ortleppi*	1 (1.8)	2 (2.9)	1 (1.1)
*E. canadensis *(G6/7)	0 (0)	13 (19.1)	6 (6.9)
*E. granulosus *s. s./*E. ortleppi *	1 (1.8)	0 (0)	0 (0)
*E. granulosus *s. s./*E. canadensis* (G6/7)	0 (0)	3 (4.4)	1 (1.1)
*E. ortleppi/E. canadensis *(G6/7)	1 (1.8)	0 (0)	0 (0)
*E. granulosus *s. s*./E. ortleppi/E. canadensis *(G6/7)	1 (1.8)	0 (0)	0 (0)
*Total*	*55*	*68*	*87*

n = number of cattle, goats, and sheep.

## Data Availability

The prevalence and genotyping data used to support the findings of this study are included in the article.
